# Bibliometric analysis of neuroscience publications quantifies the impact of data sharing

**DOI:** 10.1093/bioinformatics/btad746

**Published:** 2023-12-09

**Authors:** Herve Emissah, Bengt Ljungquist, Giorgio A Ascoli

**Affiliations:** Bioinformatics Program, College of Science, George Mason University, Fairfax, VA 22030, United States; Center for Neural Informatics, Structures, & Plasticity (CN3) and Bioengineering Department, College of Engineering & Computing, George Mason University, Fairfax, VA 22030, United States; Center for Neural Informatics, Structures, & Plasticity (CN3) and Bioengineering Department, College of Engineering & Computing, George Mason University, Fairfax, VA 22030, United States; Bioinformatics Program, College of Science, George Mason University, Fairfax, VA 22030, United States; Center for Neural Informatics, Structures, & Plasticity (CN3) and Bioengineering Department, College of Engineering & Computing, George Mason University, Fairfax, VA 22030, United States

## Abstract

**Summary:**

Neural morphology, the branching geometry of brain cells, is an essential cellular substrate of nervous system function and pathology. Despite the accelerating production of digital reconstructions of neural morphology, the public accessibility of data remains a core issue in neuroscience. Deficiencies in the availability of existing data create redundancy of research efforts and limit synergy. We carried out a comprehensive bibliometric analysis of neural morphology publications to quantify the impact of data sharing in the neuroscience community. Our findings demonstrate that sharing digital reconstructions of neural morphology via NeuroMorpho.Org leads to a significant increase of citations to the original article, thus directly benefiting authors. The rate of data reusage remains constant for at least 16 years after sharing (the whole period analyzed), altogether nearly doubling the peer-reviewed discoveries in the field. Furthermore, the recent availability of larger and more numerous datasets fostered integrative applications, which accrue on average twice the citations of re-analyses of individual datasets. We also released an open-source citation tracking web-service allowing researchers to monitor reusage of their datasets in independent peer-reviewed reports. These results and tools can facilitate the recognition of shared data reuse for merit evaluations and funding decisions.

**Availability and implementation:**

The application is available at: http://cng-nmo-dev3.orc.gmu.edu:8181/. The source code at https://github.com/HerveEmissah/nmo-authors-app and https://github.com/HerveEmissah/nmo-bibliometric-analysis.

## 1 Introduction

Omics and structural biology have benefited enormously from the consistent practice of data sharing, with thriving research subfields fueled by seminal discoveries entirely based on publicly available datasets, and vibrant ecosystems of related scientific tools (Field *et al.* 2009, Chervitz *et al.* 2011, Wilson *et al.* 2021). Neuroscience has followed suit only more recently and more gradually, in part due to greater data heterogeneity and the lack of a clear functional code akin to that of genomic sequences (Gardner *et al.* 2003, Gleeson *et al.* 2017, Poline *et al.* 2022). One particular domain of neuroscience, digital reconstructions of neural morphology, is especially amenable to data sharing (Ascoli 2006, 2015, Ascoli *et al.* 2017).

The accelerating development of advanced technologies in microscopic imaging and computational processing has greatly enhanced 3D neural reconstruction methods, enabling the creation of ever larger amounts of digital tracing data (Liu *et al.* 2022, Manubens-Gil *et al.* 2023). Capitalizing on this growth requires effective data accessibility to propel scientific discovery in neuroscience. Indeed, this is the goal of NeuroMorpho.Org, an open-access archive of 3D neural reconstructions and associated metadata (Ascoli *et al.* 2007). Today, this resource comprises hundreds of thousands of downloadable reconstructions, each of them linked to peer-reviewed publications from laboratories worldwide (Akram *et al.* 2018). Global collaborative efforts and data sharing from multiple sources are extremely valuable to researchers to gain a better understanding of the brain and its cellular constituents given the strong association between neuronal form and function (Parekh and Ascoli 2015). It is essential to determine, however, the effective extent and impact of free data exchange.

Previous research quantified the benefits of data sharing to the original authors who shared data, in addition to the data users and the community at large, in specific disciplines such as cancer microarray clinical trials (Piwowar *et al.* 2007) and noninvasive human brain imaging (Milham *et al.* 2018). However, it is not yet known whether these findings generalize to other fields, and in particular if neural morphology data sharing provides a positive return on investment for the original data owners and/or significantly impacts scientific throughput.

Here, we present a comprehensive bibliometric analysis of published literature pertaining to neural morphology to assess the impact of data sharing on the overall field as well as on individual investigators. We further introduce a dynamic web-based research tool to determine the scientific impact of uniquely identified, shared neural morphology datasets. The application serves as a valuable resource for neuroscientists to demonstrate the direct and indirect benefits of sharing their data.

## 2 Materials and methods

This study relies on datasets retrieved from NeuroMorpho.Org, Semantic Scholar, and Europe PubMed Central (EuropePMC). Semantic Scholar is an Artificial Intelligence-powered engine for research literature including a large neuroscience collection (Jones 2015). EuropePMC is an open-access archive of life science publications (Ferguson *et al.* 2021). We have selected these databases due to their extensive full-text record coverage and accessibility via Application Program Interface (API). In particular, NeuroMorpho.Org tallies availability and reusage of neural morphology data, while Semantic Scholar and EuropePMC track peer-reviewed citations, broadly considered an expedient proxy for scientific impact.

We refer here to publications that generated new digital reconstructions of neural morphology as ***Describing***. NeuroMorpho.Org divides Describing publications into three categories depending on whether the underlying datasets are publicly available (***Sharing***), unavailable (***Unsharing***), or determining availability. The database curators determine this information through direct interaction with data owners (Maraver *et al.* 2019) and update it publicly every month (neuromorpho.org/LS_queryStatus.jsp? status=Available&page=0).

NeuroMorpho.Org also tracks the publications that cite the Describing articles and/or utilize the corresponding downloaded digital reconstructions, referred to as ***Citing*** and ***Using***, respectively (neuromorpho.org/LS_usage.jsp). We fetch the Describing, Citing and Using metadata via the NeuroMorpho.Org API (neuromorpho.org/apiReference.html#literature) using a Python application, implemented with the Flask framework and released open source (https://github.com/HerveEmissah/nmo-bibliometric-analysis), to populate a MongoDB database. We also retrieve from the NeuroMorpho API (neuromorpho.org/api/neuron) and store in the database the upload date for each dataset.

We then fetch citations to and references of Describing and Using/Citing publications programmatically for storage in the MongoDB database. Specifically, the Semantic Scholar API (api.semanticscholar.org/v1/paper/{doi}) returns a JSON formatted response containing both citations and references metadata for a given publication. EuropePMC, in contrast, exposes distinct API endpoints for citations (ebi.ac.uk/europepmc/webservices/rest/MED/{pmid}/citations) and references (ebi.ac.uk/europepmc/webservices/rest/MED/{pmid}/references). We use a union of citations and a union of references from both Semantic Scholar and EuropePMC to provide a more complete record.

## 3 Results

**Figure 1.**
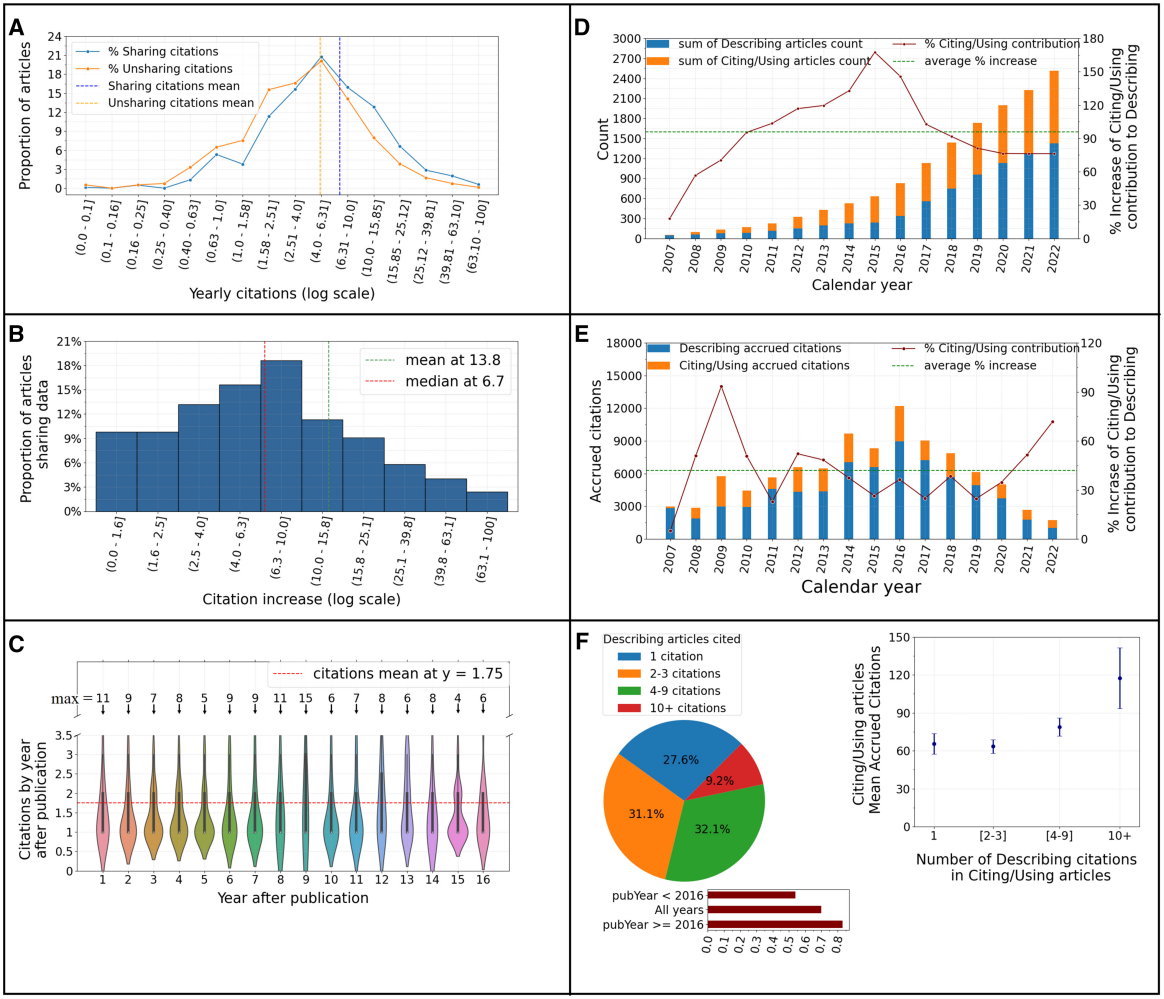
Publicly sharing digital reconstructions of neural morphology increases the number of citations to the Describing article. (A) Distributions of citations for *Sharing* and *Unsharing* articles bin-grouped using logarithmic scale. (B) Relative increase of citations to *Sharing* articles specifically due to *Using/Citing* publications. (C) Yearly citations to *Sharing* article by *Using/Citing* publications as a function of the time elapsed since the publication of the *Sharing* article. (D) Cumulative sum of *Describing* and *Using/Citing* article counts by year. (E) Citations accrued by *Describing* and *Using/Citing* articles by year. (F) Proportion of *Using/Citing* publications relying on different numbers of *Describing* articles. Bottom: ratio between the number of *Using/Citing* publications relying on ≥4 *Describing* articles and those relying on ≤3 *Describing* articles. Right: Mean number of citations accrued by *Using/Citing* publications as a function of the number of *Describing* articles cited. The error bars indicate a 95% confidence interval.

We first investigated whether openly sharing via NeuroMorpho.Org the digital reconstructions of neural morphology described in an article increases the number of citations to that article (Fig. 1). We started by comparing the number of citations to *Sharing* (*N* = 1656) and *Unsharing* (*N* = 3089) articles. Specifically, we normalized the yearly number of citations for a given *Describing* article by dividing its accrued citations by the number of years elapsed since publication. The analysis (Fig. 1A) demonstrates a significant difference in yearly citations between groups (*Sharing*: 8.91 ± 14, *Unsharing*: 6.19 ± 12; effect size +43.9%, *P* = 0.006).

We then asked whether this increase was specifically due to the citations by the Citing and Using publications. Thus, we calculated the **Citation Increase** for each Sharing article based on the following formula: **CitationIncrease = NMO_Citations/(Citations_since_upload-NMO_Citations)**, where **NMO_Citations** represents the citations to the *Sharing* article by the *Using/Citing* publications, and **Citations_since_upload** represents the overall citations to the *Sharing* article since the upload date of the corresponding dataset. The resultant histogram distribution (Fig. 1B) reveals that the Citation Increase of *Sharing* articles due to the secondary publications (13.8%) explains less than a third of the difference in citations between *Sharing* and *Unsharing* articles. Taken together, these analyses indicate that sharing neural reconstruction data through NeuroMorpho.Org increases the impact of the original publication.

Next, we explored if the numbers of citations to *Sharing* articles due to secondary publications decreases over time after publication. The results suggest a broadly uniform citation likelihood without a tendency to decrease over the whole 16 years of the project activity (Fig. 1C).

To help assess the impact of shared data, it is also interesting to compare the number of *Describing* and *Using/Citing* publications and their respective citations. Both the cumulative number of *Describing* articles and of *Using/Citing* articles increased consistently from the project launch to present (Fig. 1D). Notably, the *Using/Citing* publications, which rely on shared data, effectively double the *Describing* data literature. Moreover, comparing the overall citations to *Describing* and *Using/Citing* articles (Fig. 1E) again demonstrates that data reusage increases the number of citations in the field by nearly 50%. These results further underscore the added impact of data sharing in neuroscience.

Moreover, we found that fewer than a third of *Using/Citing* studies only refer to a single *Describing* article, and approximately the same proportion relies on 2–3 data sources (Fig. 1F). In contrast, more than 40% of *Using/Citing* articles rely on four or more sources, and nearly 10% require ten or more *Describing* publications. Interestingly, the ratio between the number of *Using/Citing* publications relying on four or more *Describing* articles and those relying on three or fewer *Describing* articles (Fig. 1F, bottom) is substantially greater after 2016 (ratio: 0.82) than before (ratio: 0.54), reflecting the increasing emphasis on big science, data aggregation, and meta-analyses. We then asked whether the *Using/Citing* articles relying on a greater number of data sources are cited more (Fig. 1F, right). Indeed, while *Using/Citing* publications relying on 1–3 data sources only accrue on average 60–65 citations, the mean number of accrued citations reaches ∼80 for articles using 4–9 sources and exceeds 110 for articles using 10 or more sources.

To provide researchers the capability to investigate the impact of *Sharing* articles on secondary publications, we made the bibliometric functionality utilized in the above analysis available as a public service through a web-based user interface (see Supplementary Materials).

## 4 Discussion

The analysis of neuroscience data sharing among researchers provides insight into current trends and raises awareness to encourage collaborations and open data release (Poldrack and Gorgolewski 2014). It is intuitively obvious that the public availability of data is beneficial to researchers who can reuse it for follow-up analysis and diverse scientific applications (Halavi *et al.* 2012). However, whether it in fact provides advantages for the data owners to share data has remained a topic for discussion.

Our findings demonstrate indeed that sharing digital reconstructions of neural morphology via NeuroMorpho.Org leads to a significant increase of citations to the original article, thus directly benefiting the authors. Moreover, the rate of data reusage remains constant for at least 16 years after sharing (the whole period analyzed), altogether nearly doubling the peer-reviewed discoveries in the field. Furthermore, the recent availability of larger and more numerous datasets fostered integrative applications, which accrue on average twice the citations of re-analyses of individual datasets. These results demonstrate the broader impact of open sharing of neural reconstructions on scientific discovery.

We also designed and deployed an open-source bibliometric tracking web-service that allows researchers to monitor reusage of their datasets in independent peer-reviewed reports. This tool can facilitate the recognition of shared data reuse for promotion and tenure considerations, merit evaluations, and funding decisions.

## Supplementary Material

btad746_Supplementary_Data
