# Supplementary material for: Bibliometric analysis of neuroscience publications quantifies the impact of data sharing
Source: Bioinformatics. 2023 Dec 9;39(12):btad746. doi: 10.1093/bioinformatics/btad746 (PMC10733721; doi:10.1093/bioinformatics/btad746)
Supplement: btad746_Supplementary_Data [file btad746_supplementary_data.docx]

The MongoDB database serves both as the repository for the bibliometric analysis (utilizing the plotting library Matplotlib for graphical visualization) and as the data source for a three-tier web-based author service, with a front-end user interface built with the React Framework and the back-end application developed in Python using Flask. The application components are built as separate Docker images exchanging information via a Docker network (Fig. S1). The bibliometric author service is deployed on a publicly accessible George Mason University server (cng-nmo-dev3.orc.gmu.edu:8181/) and is released open source as well (https://github.com/HerveEmissah/nmo-authors-app).

| 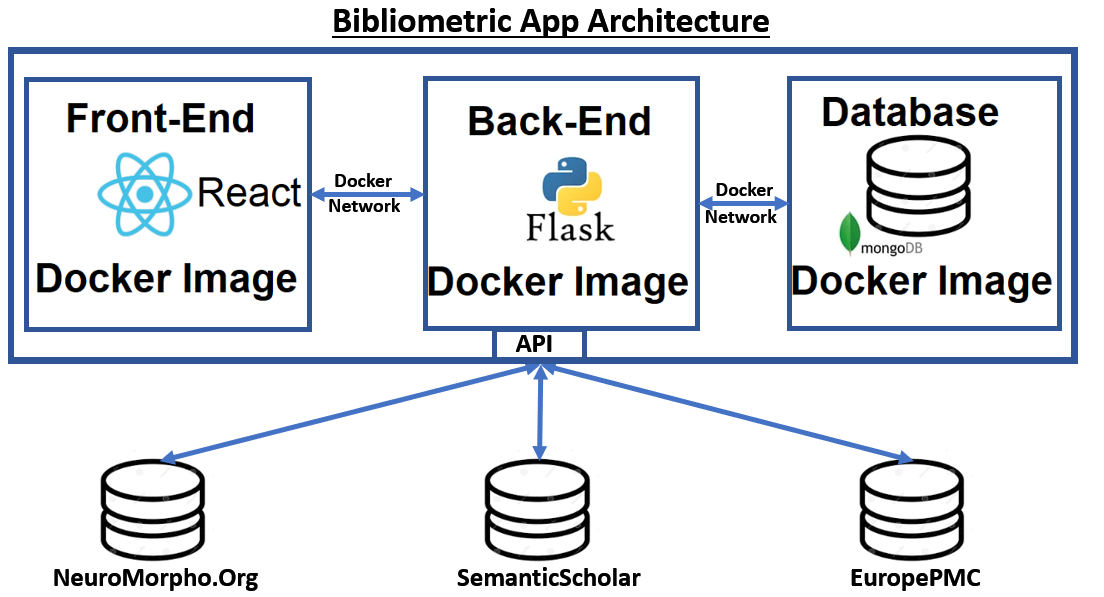  **Figure S1:** High-level schematic of the bibliometric author service 3-tier architecture, with a front-end presentation tier, Flask application, and MongoDb database. |
| --- |

To provide researchers the capability to investigate the impact of *Sharing* articles on *Using/Citing* publications, we made the bibliometric functionality utilized in the analysis described in this article available as a public service through a web-based user interface (Fig. S2). The service fetches the list of *Sharing* publications from the NeuroMorpho.Org datastore and renders the corresponding unique identifiers (PMID/DOI) on the application interface, allowing the user to select interactively the publication(s) of interest for the retrieval of the *Using/Citing* references (Fig. S2A). Moreover, the service provide real-time information on the number of times the corresponding reconstruction dataset has been downloaded from NeuroMorpho.Org. The Get Alert function enables subscribing to automated email notifications when the dataset from an article of interest is cited or reused (Fig. S2B) and allows users to unsubscribe whenever desired.

| 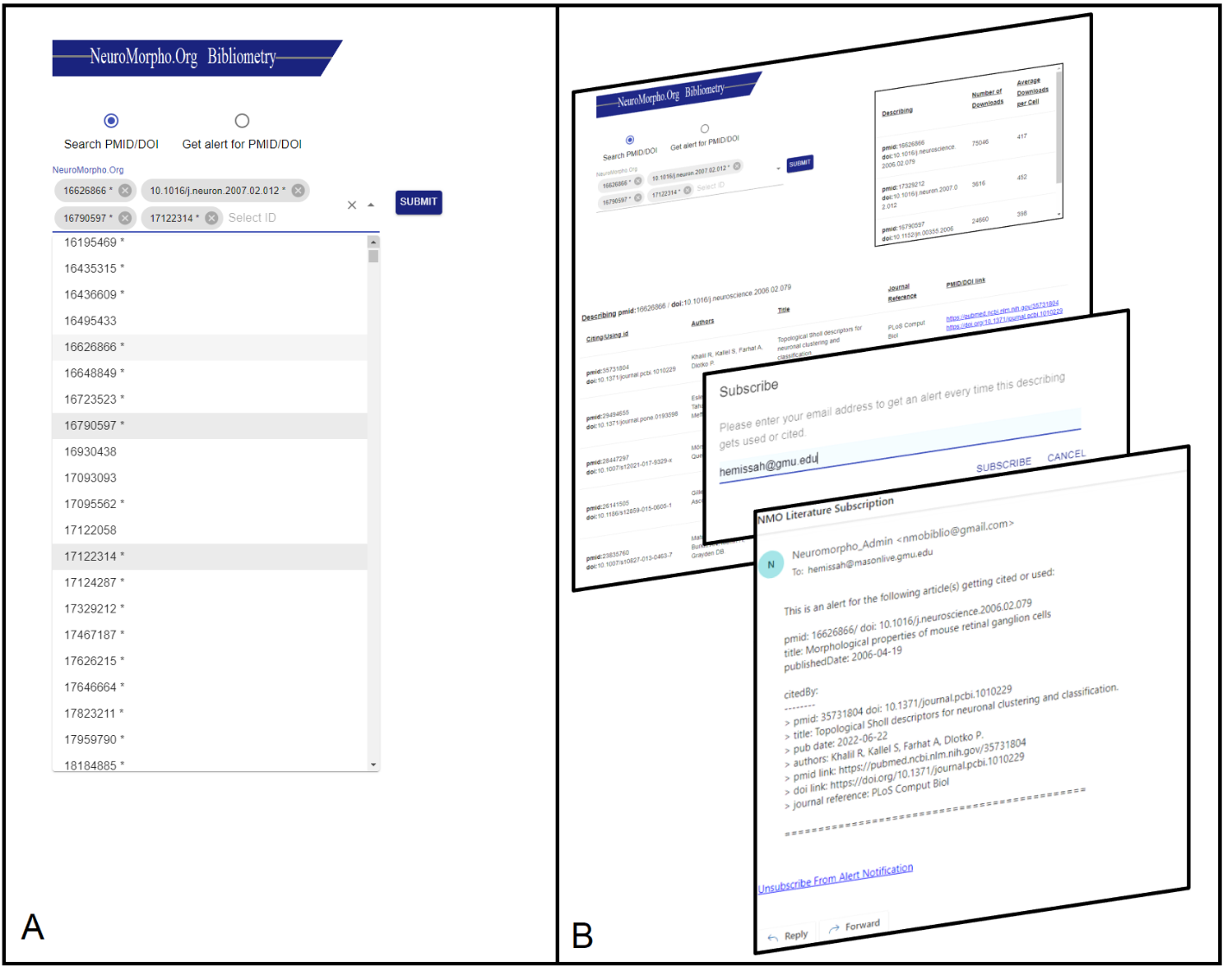  **Figure S2:** Bibliometric Author Service. **A.** The user interface lists all *Describing* publications, allowing one or more to be selected for analysis of subsequent usage or subscription to alert notification. **B.** Display of bibliographic details providing citations and dataset usage pertaining to the selected article. Additionally, when subscribing to citation alerts for specific publications, users receive email notifications when datasets from their publications of interest are reused or cited. |
| --- |
